# The basics of phosphate metabolism

**DOI:** 10.1093/ndt/gfad188

**Published:** 2023-09-02

**Authors:** Carsten A Wagner

**Affiliations:** Institute of Physiology, University of Zurich, Zurich, Switzerland

**Keywords:** bone, cell metabolism, endocrine regulation, intestine, kidney

## Abstract

Phosphorus is an essential mineral that is, in the form of inorganic phosphate (Pi), required for building cell membranes, DNA and RNA molecules, energy metabolism, signal transduction and pH buffering. In bone, Pi is essential for bone stability in the form of apatite. Intestinal absorption of dietary Pi depends on its bioavailability and has two distinct modes of active transcellular and passive paracellular absorption. Active transport is transporter mediated and partly regulated, while passive absorption depends mostly on bioavailability. Renal excretion controls systemic Pi levels, depends on transporters in the proximal tubule and is highly regulated. Deposition and release of Pi into and from soft tissues and bone has to be tightly controlled. The endocrine network coordinating intestinal absorption, renal excretion and bone turnover integrates dietary intake and metabolic requirements with renal excretion and is critical for bone stability and cardiovascular health during states of hypophosphataemia or hyperphosphataemia as evident from inborn or acquired diseases. This review provides an integrated overview of the biology of phosphate and Pi in mammals.

## INTRODUCTION

Phosphorus was the first chemical element to be identified. In 1669, Hennig Brand discovered elementary phosphorus when searching for the philosopher's stone in urine. It took more than a century until Justus von Liebig realized that inorganic phosphate (Pi) is required by plants to grow, leading to the first industrial fertilizer containing nitrogen, potassium and phosphate, called ‘super-phosphate’. Nowadays, phosphorus is used for a myriad of chemical reactions and products, including many food items, raising growing concerns about the limited natural resources of phosphorus and increasing efforts are being made to recapture phosphorus from sewage.

Phosphorus is an essential element for life on earth across all kingdoms of life. To date, no organism has been identified that can live without phosphorus. As elementary phosphorus is highly reactive with oxygen *in vivo*, phosphorus is present in organic or inorganic phosphate (Pi) molecules as PO_4_^3^^−^ ion.

This review briefly summarizes our current understanding of the functions, regulation and relevance of phosphate for human health and disease.

## PHOSPHORUS IS NOT PHOSPHATE

The terms phosphorus and phosphate are often used interchangeably, however, there are important differences. Phosphorus refers to the chemical element, while phosphate refers to molecules containing the ion PO_4_^3^^−^. The difference is important for several reasons, including the calculation of balance and chemical and metabolic reactivity. Phosphorus is highly reactive with oxygen and does not exist as such in the human body. After reaction with oxygen and water, phosphorus becomes phosphoric acid that, after dissociation and association with cations, can form various inorganic salts. Likewise, PO_4_^3^^−^ may form ester bonds typical of organic phosphate molecules (organophosphates) such as phytate, which is present in plants.

To make matters more complex, the term phosphorus is often used in clinical chemistry and dietary studies. Phosphorus-containing molecules in biological fluids or food items have different molecular weights depending on the complex and on pH. In order to calculate balances, the phosphorus content of the fluid/complex is measured or calculated and used. Important examples are dietary studies and determination of phosphorus in faeces or urine (where pH can change), while measurements in blood mostly refer to inorganic phosphate (with stable pH). When assessing the phosphorus load coming with drug formulations, care should be taken to discern phosphorus from phosphate salts (which are usually used).

## ESSENTIAL MINERAL FOR CELLULAR METABOLISM

**Figure 1:**
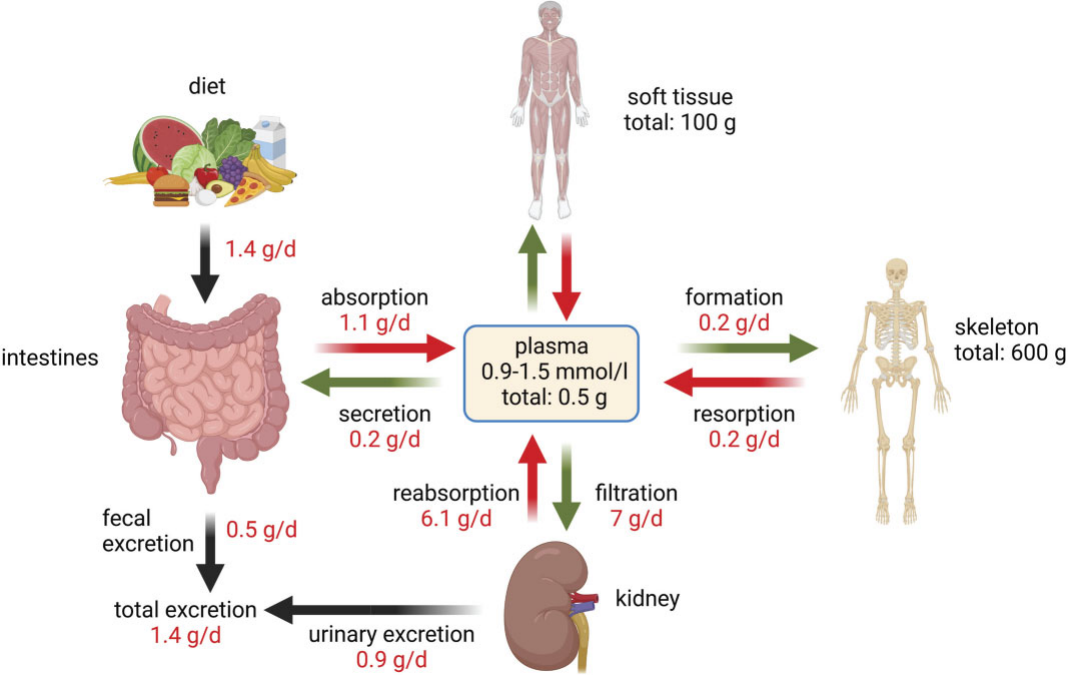
Daily phosphate fluxes. Average dietary intake of phosphorus is ≈1.4 g, of which ≈0.9 g are net absorbed by the intestine. Absorbed Pi enters the plasma pool, which is in constant exchange with soft tissue and bone. Blood also undergoes constant filtration and kidneys reabsorb 80–90% of filtered Pi. Net renal excretion plus faecal excretion matches daily intake of Pi in subjects in Pi balance. Numbers are average numbers for a 70-kg adult person on a mixed standard diet.

**Figure 2:**
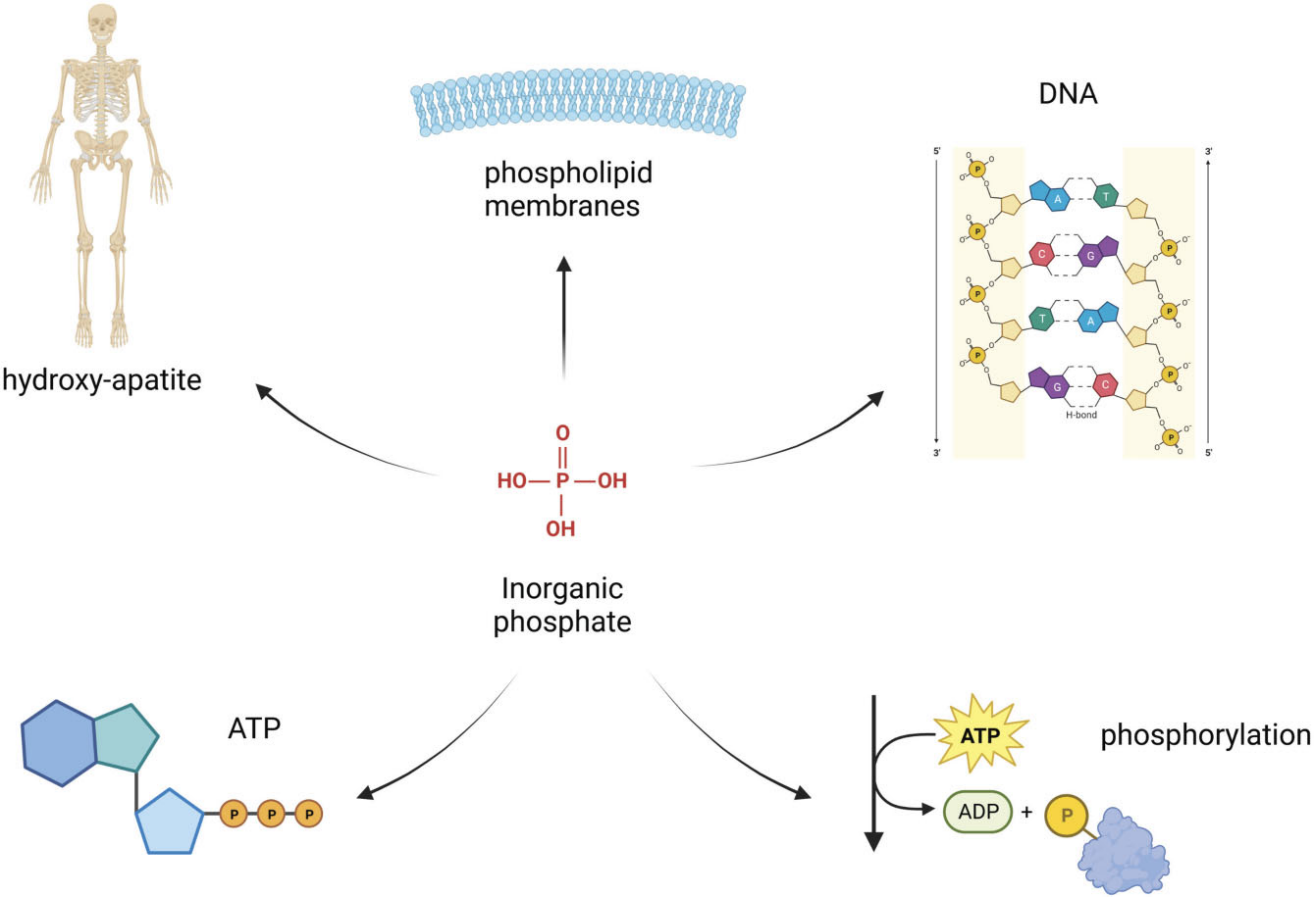
Functions of phosphate. Pi is a building block for phospholipids in biological membranes, for nucleotides in DNA and RNAs, to form ATP, is involved in intracellular signaling events and is critical for the stability of bone under the form of hydroxyapatite.

Total body phosphorus in a 70-kg person is ≈700–800 g, 80–85% of which is stored in the skeleton and the remaining 15–20% is in soft tissues and blood (5%) (Fig. 1). Pi is a building block for phospholipids, which are an integral part of all plasma and intracellular membranes, forms part of nucleotides in DNA and RNA and is required for cellular energy metabolism (Fig. 2). Pi is necessary for several enzymatic processes, including glycolysis, ammoniagenesis and oxidative phosphorylation. Phosphorylation of glucose is the first step of glycolysis and Pi is contained in adenosine triphosphate (ATP), guanosine triphosphate and uridine triphosphate. In addition, it influences the oxygen-carrying capacity of haemoglobin by its availability for 2,3-diphosphoglycerate synthesis. ATP is also essential for phosphorylation-dependent signalling events.

The overall intracellular concentration of organic phosphates has been estimated to range between 5 and 70 mmol/l, while inorganic Pi values range from 0.7 to >2 mmol/l. These differences can be explained in part by the rapid turnover between different pools of inorganic and organic phosphates. However, different techniques also yield different values; phosphorus-31 nuclear magnetic resonance found ≈0.7 mmol/l, while chemical methods found ≈2 mmol/l [1]. Of note, in patients on dialysis, rapid changes in intracellular Pi have been reported between dialysis sessions [2]. Nevertheless, intracellular Pi participates in pH buffering together with proteins and the carbon dioxide–bicarbonate metabolon. Whether there is intracellular storage of Pi in the form of polyphosphates has been discussed, but no evidence was found, while recently an intracellular polyphosphate storage organelle has been identified in *Drosophila* [3]. Clearly this field requires further examination to better understand the fate of intracellular phosphates and the role of the intracellular compartment when buffering extracellular fluctuations in Pi.

## SYSTEMIC FUNCTIONS OF PHOSPHATE

Pi concentrations in the extracellular space are ≈0.8–1.4 mmol/l (3–4.5 mg/dl). About 85% of Pi in the extracellular space is free, while 10% is protein bound and another 5% is complexed with calcium or magnesium. Extracellular Pi oscillates during the day, with its nadir between 8 and 11 a.m. [4]. The levels of organic phosphates in blood are not routinely measured and their concentrations are poorly defined.

Systemically, Pi contributes to pH buffering in extracellular fluids even though this contributes only ≈5% to the total buffer capacity.

## INTESTINAL ABSORPTION

**Figure 3:**
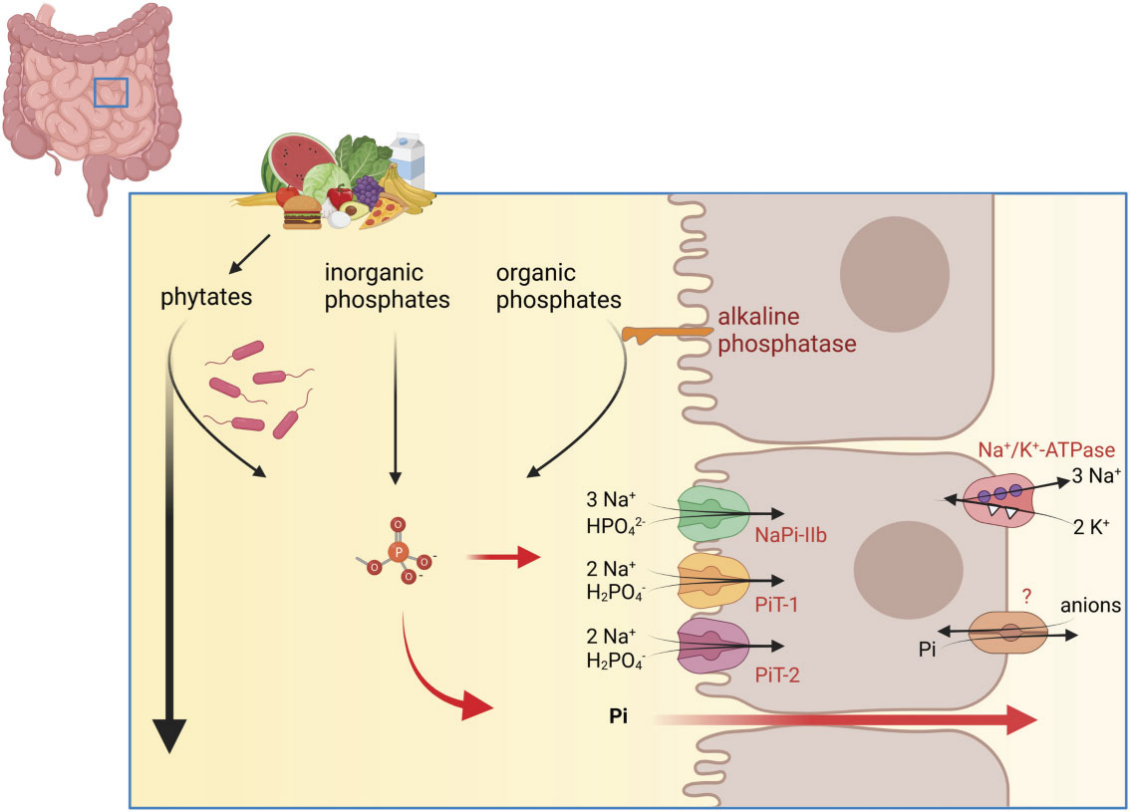
Intestinal absorption of Pi. Phosphate is delivered by diet either as inorganic salt or as organic phosphates that can be cleaved by intestinal alkaline phosphatases or by bacteria-derived phytases. Active absorption is mediated by Na^+^-dependent Pi transporters NaPi-IIb, PiT1 and PiT2 while passive absorption proceeds via the paracellular route.

Phosphate in the diet comes essentially in two different forms, as organic or inorganic phosphate [5, 6]. Organic phosphates differ in their composition depending on their origin: plant-derived organic phosphate is mostly in the form of phytates, while animal-derived organic phosphate is bound to protein. Pi salts added as food additives are the most important form of inorganic phosphate. These differences are very important for bioavailability of phosphate (Fig. 3). Plant-derived phytic acid/phytate is poorly absorbed. The human intestine has very low phytase activity to release Pi from these molecules. This phytase activity comes mostly from some bacteria (e.g. *Escherichia coli* and bifidobacteria) and yeast (e.g. various forms of aspergilli and saccharomycetes) in the gut microbiome [7]. Total phytase activity in the human intestine may adapt to phytate intake [6, 8]. However, the overall contribution of Pi from phytates to the human Pi supply remains to be established, but current trends in diets shifting to more plant-based nutrition will increase dietary phytate intake in the general population [9]. Phytates in the diet interact with cations such as calcium and positively charged trace elements such as iron and zinc and can lead to their reduced absorption.

Animal-derived organic phosphates are mostly released after cleavage of molecules by digestive enzymes, also involving membrane-bound intestinal alkaline phosphatases [6, 10]. Food additives contain various Pi salts (e.g. phosphoric acid; sodium, potassium or magnesium salts; and modified starches). The main food categories containing substantial amounts of these food additives are highly processed food items such as unflavoured pasteurized and sterilized (including ultra-high temperature) milk, infant and follow-on formulae for infants and ultra-high temperature milk, bread and rolls and meat products for adolescents and adults. In the European Union, between 500 mg and 20 g of such food additives are allowed per 1 kg of food.

Absorption of Pi in the intestine occurs through two distinct pathways: transcellular by active transport and paracellular by mostly passive driving forces [6, 11]. Active transcellular transport is mediated by a set of sodium-dependent Pi transporters located in the luminal membrane of enterocytes in the small intestine. At least three different transporters have been identified: PiT1 (SLC20A1), PiT2 (SLC20A2) and NaPi-IIb (SLC34A2) (Fig. 3). Their segmental expression varies between species: in mice, NaPi-IIb is mostly located in the distal small intestine, while in rats and humans, NaPi-IIb is located in duodenum and jejunum [6]. The exact expression of PiT1 and PiT2 has not been detailed to date. The basolateral exit pathway of Pi from enterocytes into the blood has not been identified on a molecular level. Evidence from Slc34a2 knock-out mice demonstrated that NaPi-IIb is mostly required during periods of low dietary Pi availability and protects bone from demineralization [12]. Indeed, this transporter has a high affinity for Pi but a low transport capacity. The role of PiT1 and PiT2 is much less defined. In the absence of NaPi-IIb, only very low transport activity is detectable and genetic deletion of Slc20a2 (PiT2) has no effect on mineral balance [13]. This may suggest only a marginal role of PiT1 and PiT2 transporters in Pi absorption. In contrast, a pan-inhibitor of PiT1/2 and NaPi-IIb decreased intestinal Pi absorption in rats more than a specific NaPi-IIb inhibitor [14]. Species differences may explain this discrepancy. The situation in the human intestine remains to be examined.

Under conditions of normal to high Pi availability, Pi absorption mostly proceeds via the paracellular route and high Pi permeabilities along the entire axis of the mouse and rat intestine have been measured [15]. Pi absorption is driven by the chemical gradient from the lumen to blood and by a lumen-negative transepithelial potential. Luminal Pi concentrations have been measured at 5–25 mmol/l, while blood Pi is in the range of 0.8–1.4 mmol/l. Paracellular Pi fluxes go through the tight junctions that are formed by claudins and associated proteins. To date, no specific claudin has been identified to be involved in Pi fluxes; a role of claudin 3 has been proposed but remains to be confirmed [16].

Intestinal Pi absorption is partly regulated. Active transport is stimulated by low dietary Pi intake, calcitriol, oestrogens and acidosis, while high dietary Pi intake, epidermal growth factor and glucocorticoids reduce active transport [6]. In contrast, paracellular Pi fluxes appear to be largely unregulated and are not influenced by dietary Pi intake or calcitriol [17].

## DEPOSITION AND DISTRIBUTION: SOFT TISSUE AND BONE

About 15% of the total phosphate content is deposited in soft tissue, mostly liver and skeletal muscle. Skeletal muscle takes up Pi from extracellular space, a process stimulated by insulin, and hypophosphataemia can cause insulin resistance. At least two Pi transporters contribute to Pi uptake, PiT1 and PiT2, which seem to have some overlapping functions. Combined genetic deletion of both transporters in mice causes severe myopathy [18].

In the liver, Pi is required for glycolysis and synthesis of phosphoproteins and lipids, and substantial amounts of Pi are secreted into gall. Liver expresses PiT1, PiT2 and NaPi-IIb Pi transporters [19] and the absence of PiT1 during development causes hepatic hypoplasia, while it improves glucose metabolism in adults [20].

PiT1 is also required for normal haematopoiesis [21]. Likewise, hypophosphataemia causes anaemia by increasing haemolysis at least in part due to ATP depletion.

Bone contains ≈85% of the total body phosphate content. One of the primary functions of phosphate in bone is its role as a key component of hydroxyapatite, the main mineral of bone. Hydroxyapatite provides strength and rigidity to bone, making it hard and durable. Furthermore, phosphate is integral in the regulation of bone metabolism. It acts as a signalling molecule for various cellular processes involved in bone remodelling. Phosphate plays a crucial role in the activation and differentiation of osteoblasts, the cells responsible for synthesizing new bone tissue. It also influences the activity of osteoclasts breaking down and resorbing old or damaged bone. The mechanisms by which osteoblasts take up phosphate to mineralize bone and by which osteoclasts release phosphate from the resorptive lacunae remain to be established. The Pi transporters from the SLC20 and SLC34 families are partly expressed in bone cells but appear to have no critical role in Pi transport [22]. Rather, PiT transporters may participate in bone Pi sensing (see below).

Osteoblasts together with osteoclasts and osteocytes secrete several hormones that link bone metabolism with systemic mineral homeostasis and energy metabolism [23]. Among these hormones are sclerostin, osteocalcin, receptor activator of nuclear factor κΒ ligand (RANKL) and fibroblast growth factor 23 (FGF23). The role and regulation of FGF23 will be briefly discussed below.

## RENAL EXCRETION AND REABSORPTION

**Figure 4:**
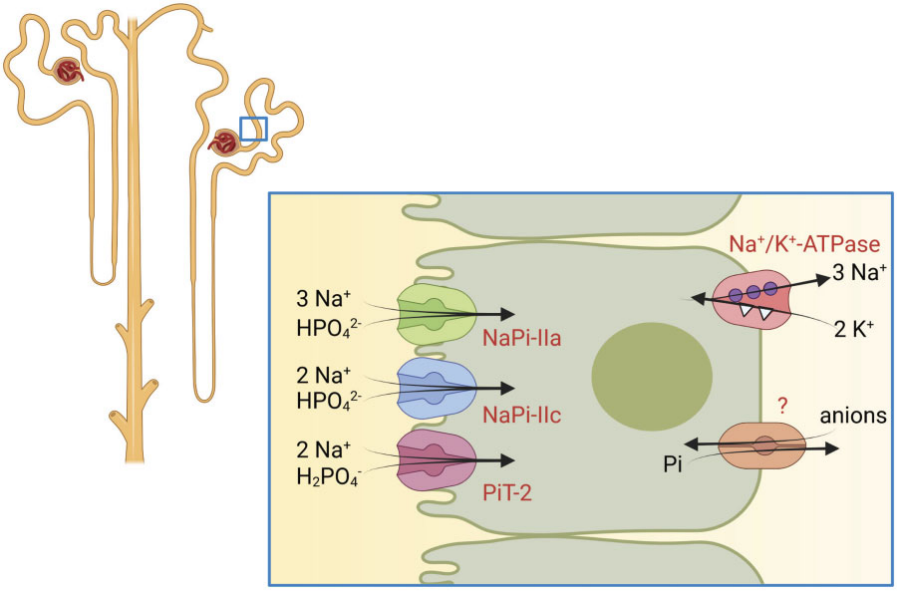
Renal reabsorption of Pi. Reabsorption of Pi in the proximal tubule is mediated by three Na^+^-dependent Pi transporters located in the luminal membrane: NaPi-IIa, NaPi-IIc and PiT2. Efflux of Pi at the basolateral membrane occurs through unknown mechanisms. Na^+^/K^+^-ATPases energize transport of Pi across the luminal membrane.

The fact that intestinal Pi absorption is mostly unregulated (except for conditions of very low Pi intake) makes the kidneys the critical gatekeeper of systemic Pi balance by regulating renal Pi excretion. Pi is freely filtered and ≈200 mmol (≈20 g) of Pi reach the primary filtrate per day. About 80% of the filtered Pi is reabsorbed in subjects on an average diet. However, renal Pi excretion can increase or decrease to almost zero under conditions of very low Pi intake. Pi reabsorption occurs mostly in the proximal tubule, even though some Pi reabsorption has been observed in distal nephron segments [11]. In the proximal tubule, at least three distinct sodium–Pi cotransporters mediate the initial step of Pi reabsorption across the luminal membrane: NaPi-IIa (SLC34A1), NaPi-IIc (SLC34A3) and PiT2 (SLC20A2) (Fig. 4) [24]. Recently, expression of NaPi-IIb has been reported but appears to be restricted to the thin and thick limb of the loop of Henle [25]. Like in the intestine, the basolateral exit pathway in proximal tubule cells remains unknown. The relevance of NaPi-IIa and NaPi-IIc for renal Pi handling is evident from genetic studies in mice and humans [24]. While in mice NaPi-IIa mediates ≈70–80% of active Pi transport and NaPi-IIc has no major relevance, in humans, inactivating mutations in *SLC34A1* and *SLC34A3* cause severe renal Pi wasting [26]. Hereditary hypophosphataemic rickets with hypercalcuria caused by *SLC34A3* mutations is associated with more severe symptoms such as rickets and persists into adulthood, while patients with *SLC34A1* mutations appear to have milder symptoms after childhood. However, *SLC34A1* mutations are associated with a high risk for chronic kidney disease (CKD), possibly due to nephrocalcinosis. Moreover, single-nucleotide polymorphisms (SNPs) in or close to the *SLC34A1* gene locus associate with a higher risk of developing CKD in several genome-wide association studies [27, 28]. The basis for this association remains unknown at this time. Likewise, SNPs in the coding and non-coding regions of *SLC34A1* associate with the risk of developing kidney stones [29].

Renal Pi handling is tightly regulated by various factors, including parathyroid hormone (PTH), FGF23 together with α-klotho, calcitriol, dopamine, growth hormone, insulin-like growth factor, thyroid hormone, hypo- and hyperkalaemia and acidosis and alkalosis [11, 24, 30]. Among these, PTH and FGF23 are the most prominent and potent regulators and will be briefly discussed below. Both hormones are phosphaturic and induce internalization and lysosomal degradation of NaPi-IIa and NaPi-IIc [31, 32].

Clinically, renal Pi handling is best assessed by measuring phosphate and creatinine in timed morning urine and blood collections in fasting subjects [33] to calculate the ratio of the renal tubular maximum reabsorption rate of phosphate to glomerular filtration rate (TmP:GFR) at the maximal stimulation of renal Pi reabsorption, avoiding the problem of circadian oscillations of plasma and urine values [34]. GFR is another major determinant of plasma Pi and this influence becomes very evident when GFR is <60 ml/min/1.73 m^2^. In subjects with an estimated GFR >60 ml/min/1.73 m^2^, age and sex influence the relationship and both must be considered [5, 35]. The range of normal TmP:GFR values differs with age and sex. In female adults it ranges between 0.8 and 1.44 mmol/l (2.0–3.6 mg/dl), while in males it ranges between 0.8 and 1.35 mmol/l (2.0–3.4 mg/dl) (for details see Payne [34]).

Urinary Pi excretion may serve as a marker for dietary Pi intake to overcome the uncertainty of dietary assessments. However, it is unclear whether the amount of Pi excreted in 24-hour urine reflects the amount of Pi absorbed during this time period, as shown for timed infusions in healthy individuals that required up to 72 hours to clear the entire Pi load [36]. Urinary 24-hour Pi excretion is even less reliable as a readout for intestinal Pi absorption in patients with CKD [37]. In balance studies, at least two consecutive 24-hour urine collections were needed to predict Pi absorption with ≥75% reliability [38]. However, it has been suggested to normalize urinary Pi to urea nitrogen in urine, yielding a better association with dietary intake [39]. Also, random spot urine samples have been evaluated to assess the correlation with 24-hour urine Pi excretion. While the correlation is weak when assessing the Pi:creatinine ratio in spot urine and 24-hour total Pi excretion, the correlation improves when age, sex and body weight are considered [40, 41].

An interesting role of phosphate has been recently shown in the bladder, where phosphate availability modulates urinary tract infections. PiT1 activity reduces intraorganellar Pi in fusiform vesicles inside the bladder epithelia, which allows uropathogenic *E. coli* to escape from these vesicles and spread [42].

## INTERPLAY OF PHOSPHATE AND CALCIUM

Pi functionally and chemically interacts with calcium. Pi and calcium easily react in biological fluids to form calcium phosphate and calcium phosphate protein particles (CPPs). This equilibrium is partly described by the calcium phosphate product (Ca × Pi). Physiologically, Ca × Pi is 2.4 mmol/l × 1.2 mmol/l = 2.88 mmol^2^/l^2^ and is considered to be elevated when >4.9 mmol^2^/l^2^. The relevance of the Ca × Pi product becomes easily evident when considering that it correlates with cardiovascular morbidity and mortality in patients [43, 44]. In the kidney, calcium phosphate crystals may trigger inflammation and CKD [45].

The uncontrolled crystallization and precipitation of calcium and phosphate is physiologically buffered by the formation of calcium protein particles (CPPs) that contain fetuin-A [46]. First, calcium phosphate reacts with monomeric fetuin-A, forming calciprotein monomers (CPMs) that then aggregate to primary CCPs that after consolidation of the calcium phosphate crystals turn into secondary CPPs [46]. The transformation of CPMs to secondary CPPs is modulated by a variety of factors, including pH, phosphate itself, magnesium and pyrophosphate. Measurement of the half-transformation (T_50_) time assesses the speed of transition, and shorter T_50_ correlates with a higher propensity of plasma to form calcifying particles. Clinically, a low T_50_ is associated in patients with various forms and stages of kidney disease with worse outcomes and in the general population with an increased risk for cardiovascular morbidity and mortality [46, 47].

An important inhibitor of ectopic tissue calcification is extracellular pyrophosphate (PPi). PPi is formed from ATP that is released from cells by the ABCC6 (formerly MRP6) transporter and is then converted into PPi and adenosine by the membrane-anchored ectonucleotidases NPP1 (encoded by ENPP1) and CD73 (NT5E). Adenosine serves as an additional inhibitor of tissue calcification by blocking the degradation of PPi to two Pi molecules by tissue non-specific alkaline phosphatase (TNAP). The relevance of this system is evidenced in patients with inactivating mutations in ABCC6 or ENPP1 and massive ectopic calcifications [48, 49]. High PPi levels are associated with disease and can cause calcium pyrophosphate depositions in joints, leading to calcium pyrophosphate dihydrate crystal-related arthropathies [50].

Another level where calcium and phosphate interact is on the endocrine level, where a network of hormones regulates not only the homeostasis of each mineral, but also the balance between them. The latter point is important to consider when studying the function of these hormones or measuring them in a diagnostic setting.

## ENDOCRINE REGULATION

**Figure 5:**
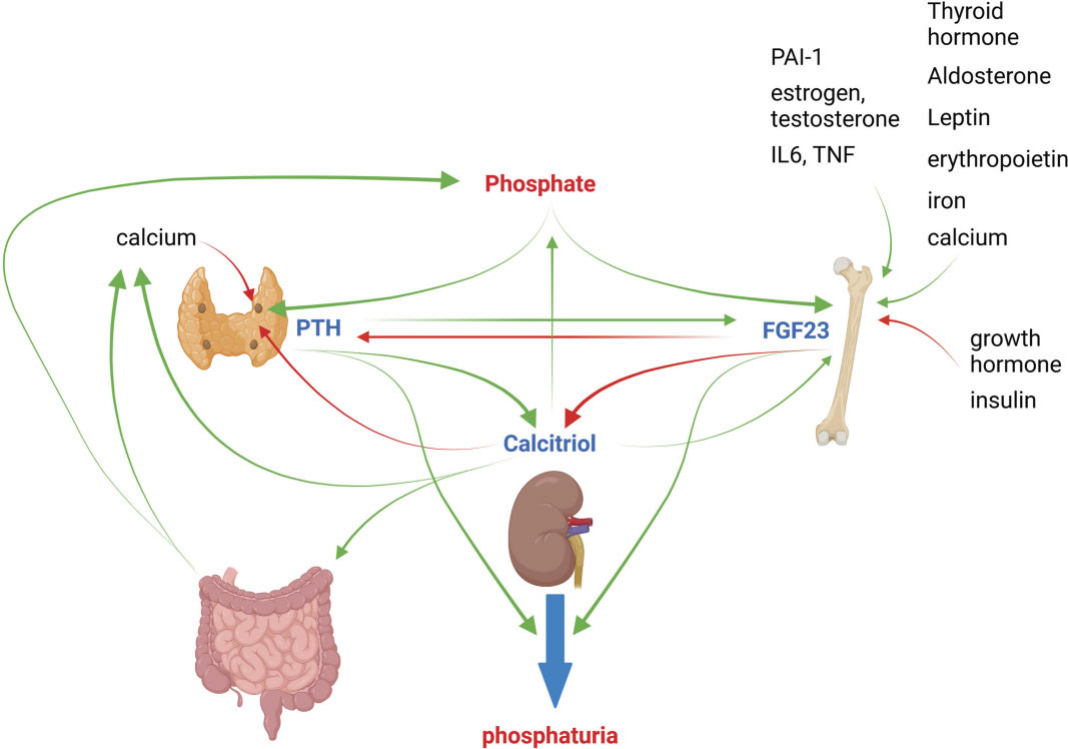
Endocrine regulation of phosphate homeostasis. The three major endocrine factors regulating phosphate homeostasis and its link to calcium metabolism. For details, see the text.

Both calcium and phosphate homeostasis are regulated by a large variety of different factors. Some of these factors specifically regulate only one mineral while key hormones impact on both minerals. The main endocrine regulators are calcitriol, PTH and FGF23 together with α-klotho. To better understand the intricate regulation of mineral homeostasis it is important to consider that the three main factors regulate each other through positive and negative feedback loops and that despite this regulation, additional factors will determine the balance between the hormones (Fig. 5). Calcium and phosphate are among these factors and thereby allow for differential regulation of mineral homeostasis.

### PTH

PTH is a master regulator of both calcium and phosphate homeostasis, increasing blood calcium levels while promoting renal Pi excretion at the same time. This dual action protects the organism from low calcium and prevents the precipitation of calcium phosphate crystals. PTH also stimulates FGF23 secretion from bone and enhances synthesis of calcitriol in the kidney. Since the action of PTH on bone releases phosphate from bone and the stimulation of intestinal calcium and phosphate absorption enhances the Pi load, PTH compensates by increasing phosphaturia due to downregulation of renal Pi transporters. Secretion of PTH from the parathyroid glands is not only stimulated by low ionized calcium, but also by high plasma Pi, which renders the calcium-sensing receptor (CaSR) less sensitive to ionized calcium [51].

Hyper- and hypophosphataemia can cause relative resistance to PTH. In experimental animals, the kidney is relatively insensitive to PTH, while this effect can be overcome with nicotinamide [52]. In patients with CKD and hyperphosphataemia, the phosphaturic effect of PTH is partly blunted. Reduced expression of PTH receptor 1 in the kidneys and bone reduces the cellular effects of PTH in its target organs, leading to a compensatory increase in PTH secretion. Simultaneously, reduced calcitriol levels and loss of α-klotho in parathyroid gland cells reduce the inhibitory effects of calcitriol and FGF23 on PTH secretion and parathyroid gland growth, leading to secondary hyperparathyroidism (sHPT). Also, it has been speculated that direct effects of high phosphate on the CaSR might contribute to sHPT [53, 54].

### FGF23

FGF23 is a major endocrine regulator of Pi homeostasis [30, 55–59]. Physiologically, FGF23 is produced mainly by osteocytes [23], but in diseased organs, FGF23 can be produced by a large variety of different cell types, including kidney cells. FGF23 secretion is stimulated upon increased Pi intake and FGF23 acts primarily on the kidneys to stimulate renal clearance of Pi and reduce circulating calcitriol levels. FGF23 downregulates the renal Pi transporters NaPi-IIa and NaPi-IIc, stimulates calcitriol degradation by CYP24A1 and suppresses calcitriol synthesis by CYP27B1 [30, 32]. FGF23 requires α-klotho to bind to its main cognate receptor FGFR1c [60]. The main stimulators of bone FGF23 secretion are PTH and calcitriol, along with leptin, several immune cytokines (tumour necrosis factor, interleukin-6), aldosterone, sex hormones, thyroid hormone, plasminogen activator inhibitor-1, iron and erythropoietin [56, 57, 61–63]. Increases in Pi and calcium also stimulate FGF23, but the molecular mechanisms responsible are unclear and secretion occurs with a delay of several hours. In contrast, growth hormone and insulin suppress FGF23 secretion [64] (Fig. 5). α-klotho acting as a scaffold for FGF23 binding to the FGFR1-3 receptors has actions independent from FGF23 that include cleavage of NaPi-IIa transporters, regulation of transient receptor potential cation channel subfamily V member 5 (TRPV5) calcium channels and modulation of renal disease [30, 65].

### Calcitriol

The classic functions of calcitriol are related to mineral homeostasis by stimulating the intestine active transcellular calcium and Pi absorption and by promoting mineralization of bone tissue [55, 66]. In the kidney, calcitriol enhances active calcium reabsorption via TRPV5 channels, while the effects on proximal tubular Pi handling are somewhat more complex. *In vitro*, calcitriol stimulates Pi transport, while *in vivo* the positive effect is counterbalanced by the stimulation of FGF23 release and the phosphaturic effects of FGF23. The effects of calcitriol are mediated by the vitamin D receptor. Under Pi-depleting conditions, renal and intestinal Pi transport are enhanced and calcitriol is elevated. However, genetic ablation of the vitamin D receptor or Cyp27b1 (the enzyme producing calcitriol) in mice does not impair the compensatory increase in Pi transport in Pi depletion [67, 68]. Calcitriol also stimulates FGF23 production in bone and suppresses PTH secretion from parathyroid glands [30, 55].

### Differential endocrine actions

Under conditions of a primary decrease in ionized calcium concentrations, an increase in PTH will not only increase renal reabsorption of calcium and release of calcium from bone, but also increases phosphate release from bone. This Pi will in turn be excreted by the kidneys due to a PTH-initiated downregulation of Pi transporters. PTH will also stimulate renal calcitriol synthesis, promoting intestinal calcium and phosphate absorption. There is also PTH-independent renal calcitriol synthesis stimulated by low calcium [66]. The effects on intestinal Pi absorption will again be counteracted by a PTH-stimulated enhanced FGF23 release that suppresses calcitriol and again stimulates renal Pi excretion. Thus, under these conditions, calcium levels can increase without increasing Pi levels.

If there is a primary increase in plasma phosphate, this will directly stimulate PTH secretion and FGF23 release from bone [55, 69]. The latter can be also independent from PTH and is sustained even if PTH returns to baseline. A strong increase in plasma phosphate levels can also lead to a decrease in ionized calcium due to complexion, which impacts PTH and calcitriol levels as in a primary reduction of calcium. The main difference between the primary calcium decrease or phosphate increase is in the sustained secretion of FGF23 as long as Pi is elevated. However, in several pathologies the combined control of calcium and phosphate by the same endocrine network can cause abnormal calcium and/or phosphate levels. This is evident in CKD as well as in primary genetic causes of hypophosphataemia such as in renal loss of phosphate due to mutations in either *SLC34A1* or *SLC34A3*, where hypophosphataemia triggers an increase in calcitriol, leading to excessive intestinal phosphate and calcium absorption with hypercalcaemia and hypercalcuria [70].

## SENSING PHOSPHATE

The mechanisms by which Pi is sensed in mammals are only now emerging. Mechanisms for sensing either extra- or intracellular Pi have been reported [71]. Among these mechanisms are the PiT1 and PiT2 transporters and the CaSR that have been implied in sensing extracellular Pi in bone and parathyroid glands, respectively [51, 72]. Intracellular Pi may be sensed by a signalling complex involving inositol hexakisphosphate kinases generating inositol hexakisphosphate (IP_6_), which serves a substrate to form IP_7_ and IP_8_ that may, via XPR1, modulate phosphate transporters and cellular Pi metabolism [73–75]. Another mechanism by which the kidney proximal tubule may sense Pi has been recently proposed: Pi is taken up by Pi transporters, stimulates glycolysis and production of glycerol-3-phosphate that stimulates bone FGF23 release [76]. Since FGF23 downregulates Pi transport and uptake, it remains unclear if this mechanism can contribute to sustained high FGF23 levels. Also, proximal tubule is not primarily glycolytic. Clearly, more research is required to expand our understanding of this essential aspect of phosphate metabolism.

## SEX AND AGE DIFFERENCES

Age and sex modulate plasma Pi levels, in part by affecting intestinal and renal Pi handling [77]. Plasma Pi levels are highest after birth and decrease continuously until the end of puberty [78]. Thereafter they remain constant in men, whereas they significantly increase in women and remain higher. Sex modulates plasma Pi and TmP:GFR in healthy humans. In mice, oestrogen downregulates renal NaPi-IIa and NaPi-IIc expression [79] while it stimulates active intestinal Pi transport via NaPi-IIb [80]. The effect of age on the sex-dependent regulation of renal and intestinal Pi transporters and the endocrine network regulating crosstalk has not been addressed in animal models or humans. The increase in blood Pi levels in women occurs in those >40–45 years, however, data on intestinal Pi absorption and renal Pi handling in postmenopausal women are missing. Age plays a major role in Pi balance. In rats, tubular Pi reabsorption is highest around weaning and then decreases [81]. Growth hormone does not directly stimulate NaPi-IIa but acts possibly via insulin-like growth factor 1 and through the suppression of FGF23 [64, 82, 83]. In rodents, NaPi-IIc expression is highest post-natally and declines thereafter [84]; in adult mice it contributes little to overall Pi reabsorption, as evident from knock-out mouse studies [85–88].

A recent study showed that male and female mice had similar blood Pi levels irrespective of age and diet, with a trend towards higher levels with higher Pi availability [89]. Females had higher FGF23 levels under all conditions and FGF23 increased more with age and high Pi availability, while PTH was elevated only in males with higher Pi availability. Pi availability had no effect on bone parameters. We also recently found no sex differences in blood Pi, but PTH was higher in male mice [90]. However, in contrast to these studies in rodents, humans exhibit differences between females and males in blood Pi levels and show an association of higher blood Pi levels in subjects with higher Pi intake. We have recently shown in 18-month-old male mice fed for 1 year a normal versus a mildly elevated Pi diet, plasma Pi levels along with calcitriol were elevated and PTH decreased and bone showed reduced bone mineral density in animals receiving higher Pi [91]. Thus this topic deserves further analysis in humans under controlled conditions.

In addition to age and sex, circadian rhythms exerts profound effects on blood Pi levels as well as on several of its endocrine regulators and renal handling of Pi [4]. Timed measurements are critical when assessing blood Pi, renal handling and endocrine regulators such as PTH, FGF23 and calcitriol. Whether circadian rhythms of mineral parameters are affected by age, sex or dietary Pi intake is unknown. However, circadian rhythms are disturbed in CKD, influencing mineral metabolism [92].

## PHOSPHATE IN DISEASE

Phosphate deficiency as well as Pi overload can be causes of severe disease manifestations. In contrast, the occurrence and role of dietary Pi overload in the general population is rather controversially discussed.

### General population

A series of epidemiological studies associated high dietary Pi intake or high-normal Pi plasma levels (within the normal Pi range) with a higher risk for incipient heart disease, vascular calcification, cardiovascular morbidity and mortality, progression of kidney disease and bone disease [93–97]. Major problems with some of these studies are that most data originate from studies conducted in US populations. Moreover, there are uncertainties in accurately assessing dietary Pi intake and Pi bioavailability and the possibility of confounding factors associated with the intake of highly processed food items. On the other hand, a few studies tested the effect of high Pi on endocrine or cardiovascular endpoints under controlled experimental conditions in healthy participants and animals. In these studies, high phosphate intake caused a higher sympathetic nerve outflow, higher systolic blood pressure or bone loss [91, 98–100]. Moreover, animal studies suggest that high Pi intake can cause left ventricular hypertrophy as well as kidney damage [45, 101]. Clearly, the health risk associated with high dietary phosphate intake must be assessed further. The European Food Safety Authority stated in their 2019 report that the acceptable daily intake is 40 mg/kg body weight/day, or 2.8 g for a 70-kg adult [102]. Notably, this value was set based on a single publication in rats and can be considered as purely hypothetical.

### Hypophosphataemia

Hypophosphataemia can be subdivided into moderate [0.3–0.65 mmol/l (1–2.5 mg/dl)] and severe [<0.3 mmol/l (<1.0 mg/dl)] forms based on plasma phosphate levels. The main symptoms can be explained on a cellular level by energy depletion due to insufficient ATP production. Symptoms include muscle weakness, including respiratory muscles with respiratory failure, bone pain and loss, neuropathies, tremors and seizures, haemolysis and anaemia, metabolic acidosis and insulin resistance [103, 104]. In children, rickets and bone deformities are prominent.

In children, inborn defects of phosphate metabolism are among the most frequent causes and can be caused by gene defects in either renal phosphate transporters or in endocrine regulators [26]. The most common form of inherited hypophosphataemia is due to mutations in the *PHEX* gene causing X-linked hypophosphataemia [103]. In adults, hypophosphataemia can be caused by reduced intestinal absorption, internal shifts or renal losses [104]. Measurement of TmP:GFR can help to identify the cause of hypophosphataemia.

### Hyperphosphataemia

Plasma Pi levels >1.46 mmol/l (4.5 mg/dl) are considered hyperphosphataemic. Common causes of hyperphosphataemia are advanced stages of CKD, acute kidney injury, phosphate supplements with or without vitamin D supplements, hypoparathyroidism, metabolic and respiratory acidosis or rhabdomyolysis and tumour lysis syndrome [5, 105–107]. Acutely, hyperphosphataemia causes hypocalcaemia, while the long-term consequences are mostly linked to inflammation, ectopic calcifications of vessels and highly increased cardiovascular morbidity and mortality [108]. For a more detailed discussion of hyperphosphataemia in CKD patients, readers are referred to a series of excellent reviews on various aspects of this problem [5, 105, 106, 109, 110].

## THERAPIES TARGETING PHOSPHATE METABOLISM

Therapies targeting phosphate metabolism alter mostly intestinal absorption and renal excretion. Some therapies target deposition in soft tissue, such as insulin/glucose infusions, or release from bone, such as bisphosphonates. In states of hypophosphataemia, phosphate supplements are the mainstay of therapy. A special case is hypophosphataemia due to FGF23 excess, such as in X-linked hypophosphataemia (XLH) or in patients with tumour-induced osteomalacia (TIO). While in TIO, removal of the FGF23-producing tumour is the primary therapy, neutralization of FGF23 in these patients, and particularly in patients with XLH, has become an important therapy modality and ameliorates many of the problems [103].

Hyperphosphataemia in CKD is a major problem and remains insufficiently controlled by current therapies [106]. These therapies include dietary control of phosphate, phosphate binders, suppression of PTH secretion with calcimimetics and dialysis to remove excess phosphate. A number of novel therapies have been discussed and are under development [11, 106]. These drugs aim to reduce intestinal phosphate absorption by blocking active or passive pathways or try to increase renal clearance of phosphate by inhibiting renal Pi transporters. Inhibition of active Pi absorption in the intestine by the NaPi-IIb transporter has produced promising results in animal studies but has completely failed in phase 1 and 2 studies. A novel pan-inhibitor of intestinal Pi transporters reduces intestinal phosphate absorption in rats; human studies are awaited [111]. Tenapanor is a non-absorbable sodium–hydrogen exchanger 3 inhibitor that indirectly reduces intestinal paracellular Pi absorption. Phase 3 studies in patients on haemodialysis have shown its potential to reduce hyperphosphataemia, particularly when combined with phosphate binders [11]. Whether it will improve cardiovascular outcomes has to be demonstrated. Inhibition of renal Pi reabsorption may reduce hyperphosphataemia or systemic Pi overload in earlier stages of CKD as long as there is sufficient renal filtration of Pi. In mice and rats, short-term application of such inhibitors reduces hyperphosphataemia, PTH and FGF23 and ameliorates vascular calcification.

## OUTLOOK

Phosphate is an essential mineral and its balance has to be tightly controlled to avoid states of hypophosphataemia with cellular energy deficiency as well as the sequelae of hyperphosphataemia with ectopic calcifications and cardiovascular disease. In times of very high consumption of phosphate in industrialized countries and evidence associating dietary phosphate with disease and mortality in the general population, as well as in the very vulnerable population of patients with CKD, a better understanding of the effects of phosphate in the human body is required. Fields that must be further addressed are the role of CPPs, transport mechanisms of phosphate in the intestine and other organs (e.g. bone), Pi sensing, the impact of Pi (and/or CPPs) on inflammation and cardiovascular health, the interactions of phosphate and phosphate-containing crystals on gut microbes and the role that phosphatotropic hormones play in these processes.

## Data Availability

No new data were generated or analysed in support of this research.
